# Conserved Mechanism of Bicarbonate-Induced Sensitization of CatSper Channels in Human and Mouse Sperm

**DOI:** 10.3389/fcell.2021.733653

**Published:** 2021-09-28

**Authors:** Juan J. Ferreira, Pascale Lybaert, Lis C. Puga-Molina, Celia M. Santi

**Affiliations:** ^1^Department of Obstetrics and Gynecology, Washington University School of Medicine, St. Louis, MO, United States; ^2^Department of Neuroscience, Washington University School of Medicine, St. Louis, MO, United States; ^3^Research Laboratory on Human Reproduction, Faculté de Médecine, Université Libre de Bruxelles, Bruxelles, Belgium

**Keywords:** sperm, CatSper channels, CAMP/PKA, capacitation, fertilization, hyperactivation, sperm motility, calcium

## Abstract

To fertilize an egg, mammalian sperm must undergo capacitation in the female genital tract. A key contributor to capacitation is the calcium (Ca^2+^) channel CatSper, which is activated by membrane depolarization and intracellular alkalinization. In mouse epididymal sperm, membrane depolarization by exposure to high KCl triggers Ca^2+^ entry through CatSper only in alkaline conditions (pH 8.6) or after *in vitro* incubation with bicarbonate (HCO_3_^–^) and bovine serum albumin (capacitating conditions). However, in ejaculated human sperm, membrane depolarization triggers Ca^2+^ entry through CatSper in non-capacitating conditions and at lower pH (< pH 7.4) than is required in mouse sperm. Here, we aimed to determine the mechanism(s) by which CatSper is activated in mouse and human sperm. We exposed ejaculated mouse and human sperm to high KCl to depolarize the membrane and found that intracellular Ca^2+^ concentration increased at pH 7.4 in sperm from both species. Conversely, intracellular Ca^2+^ concentration did not increase under these conditions in mouse epididymal or human epididymal sperm. Furthermore, pre-incubation with HCO_3_^–^ triggered an intracellular Ca^2+^ concentration increase in response to KCl in human epididymal sperm. Treatment with protein kinase A (PKA) inhibitors during exposure to HCO_3_^–^ inhibited Ca^2+^ concentration increases in mouse epididymal sperm and in both mouse and human ejaculated sperm. Finally, we show that soluble adenylyl cyclase and increased intracellular pH are required for the intracellular Ca^2+^ concentration increase in both human and mouse sperm. In summary, our results suggest that a conserved mechanism of activation of CatSper channels is present in both human and mouse sperm. In this mechanism, HCO_3_^–^ in semen activates the soluble adenylyl cyclase/protein kinase A pathway, which leads to increased intracellular pH and sensitizes CatSper channels to respond to membrane depolarization to allow Ca^2+^ influx. This indirect mechanism of CatSper sensitization might be an early event capacitation that occurs as soon as the sperm contact the semen.

## Introduction

After ejaculation, human and mouse sperm cannot fertilize an egg until they undergo a maturation process in the female genital tract known as capacitation (Austin, 1951, 1952; Chang, 1951). Capacitation induces two phenotypic changes in sperm. First, they become hyperactive, in which the frequency and pattern of flagellar beating changes to help sperm detach from the oviduct walls and penetrate the egg (Yanagimachi, 1970; Demott and Suarez, 1992; Suarez et al., 1993). Second, capacitated sperm undergo regulated exocytosis of the acrosomal content, releasing enzymes that help the sperm penetrate the zona pellucida surrounding the egg and exposing the sperm sites that will fuse with the egg (Stival et al., 2016; Hirohashi and Yanagimachi, 2018).

Capacitation involves several molecular events such as hyperpolarization of the plasma membrane (Zeng et al., 1995; Santi et al., 2010; de la Vega-Beltran et al., 2012; Chavez et al., 2013; López-González et al., 2014) and increases in intracellular pH, cyclic AMP (cAMP) concentration, protein kinase A (PKA) activity, tyrosine phosphorylation (Visconti et al., 1995; Battistone et al., 2013; Puga Molina et al., 2018), and intracellular calcium concentration ([Ca^2+^]_*i*_) (Baldi et al., 1991; Suarez et al., 1993; Breitbart, 2002a,b; Luque et al., 2018). Some of these events take place as soon as as the sperm are ejaculated, whereas others occur over a longer period of time in the female tract or in a medium that support *in vitro* capacitation (Visconti, 2009).

The increase in [Ca^2+^]_*i*_ during capacitation occurs via activity of the sperm-specific Ca^2+^ channel, CatSper (Ren et al., 2001; Carlson et al., 2003; Xia et al., 2007; Strunker et al., 2011; Alasmari et al., 2013; Smith et al., 2013; Chavez et al., 2014; Vyklicka and Lishko, 2020), which is activated by both membrane depolarization and alkalinization (Kirichok et al., 2006). However, some data have suggested that how CatSper is activated in response to membrane depolarization differs between mouse and human sperm (Lishko et al., 2011). In experiments with mouse sperm, membrane depolarization induced Ca^2+^ entry through CatSper channels only in alkaline media or after *in vitro* capacitation (incubation with bicarbonate and bovine serum albumin) (Carlson et al., 2003; Chavez et al., 2014). Conversely, in experiments with human sperm, membrane depolarization triggered [Ca^2+^]_*i*_ increase in neutral media and in non-capacitated conditions. These differences may reflect species-specific differences in CatSper regulation. Alternatively, these differences could be due to the fact that most of the mouse experiments were done with epidydimal sperm, which have not been exposed to semen, whereas most of the human experiments were done with ejaculated sperm, which have been exposed to semen.

Here, we aimed to determine the mechanism(s) by which CatSper is activated in mouse and human sperm. Our findings indicate that CatSper is activated similarly in mouse and human epididymal sperm and is activated similarly in mouse and human ejaculated sperm. Specifically, we provide evidence that – in both mouse and human sperm – exposure to bicarbonate (HCO_3_^–^), which is a major semen component, activates the soluble adenylyl cyclase/PKA pathway and increases intracellular pH. This increase in intracellular pH sensitizes CatSper to increase [Ca^2+^]_*i*_ in response to membrane depolarization.

## Materials and Methods

### Mice

All mouse procedures were reviewed and approved by the Institutional Animal Care and Use Committee of Washington University in St. Louis (St. Louis, MO, United States) (protocol # 20-0126) and were performed according to the National Institutes of Health Guiding Principles for the care and use of laboratory animals. *CatSper* knock-out mice (in which the second exon encoding the first putative transmembrane domain was replaced with an IRESLacZ sequence followed by a neomycin resistance gene in 129S4/SvJae-derived J1 ES cells, Ren et al. (2001) were obtained from Jackson Laboratory. C57BL/6 males were used as wild-type controls. Soluble adenylyl cyclase (sAC) knock-out mice were generously provided by Lonni Levin and J Buck (Buck et al., 1999; Hess et al., 2005).

### Human Participants

This study conformed with the Declaration of Helsinki (except for registration in a database) and was approved by the Washington University in St. Louis Institutional Review Board (protocol #201706077). All subjects signed written informed consent forms approved by the Washington University in St. Louis Institutional Review Board.

### Preparation of Mouse Caudal Epididymal Sperm

Adult (60–90 days old) males were euthanized by cervical dislocation, and the cauda epididymis was removed and incubated in non-capacitated or capacitated HS media (defined below), pH 7.4, for 15–20 min or 90 min, respectively, at 37^*o*^C. After incubation, the motile sperm were removed for use in experiments.

### Preparation of Mouse Ejaculated Spermatozoa

Mouse ejaculated spermatozoa were isolated as described previously (Li et al., 2015). Briefly, each adult male (60–90 days old) was placed in a cage with a female in estrus before sunrise (3–5 am). Mice were watched under a red light to determine when coitus occurred. Five to ten minutes after coitus was complete, the females were euthanized, and their uteri dissected out. Sperm were retrieved by washing the uterus with 500 μl of non-capacitated HS media, yielding an average of 10.64 ± 6.85 (SD) million sperm per milliliter. Ejaculated mouse sperm did not appear to be capacitated, as the percentages of hyperactivated and acrosome-reacted sperm were similar to the percentages in non-capacitated epididymal sperm (data not shown).

### Preparation of Human Epididymal Sperm

Small (1 cm) vas deferens pieces were obtained from healthy donors undergoing elective vasectomy procedures under local anesthesia. Specimens were kept in non-capacitated HS media at room temperature and flushed within an hour after surgery with non-capacitated recording solution (described below) within 1 h of obtaining the sample.

### Preparation of Human Ejaculated Sperm

Freshly ejaculated semen samples were obtained by masturbation after two days of abstinence from healthy donors with normal semen parameters as defined by the World Health Organization (≥32% progressive motility; ≥40% total motility; ≥15 × 10^6^ cells/ml). Within two hours, sperm were purified by the swim-up technique at 37°C in a VWR Borosilicate Glass tube with 2 ml of non-capacitated recording solution and 1 ml of sample at the bottom. Sperm were allowed to swim out of the semen for an hour, then collected in recording solution. Human sperm were capacitated by adding 25 mM HCO_3_^–^ (Sigma-Aldrich) and 5 mg/ml BSA to the non-capacitating recording solution and incubating the cells for 18–24 h at 37^*o*^C and 5% CO_2_.

### Ca^2+^ Imaging

After swim up, motile sperm were incubated with 2–4 μM Fluo-4 AM (catalog number F-4201, Life Technologies) and 0.05% Pluronic F-127 (catalog number P3000MP, Life Technologies) in HS media at 37°C for 45 min (human sperm) or 90 min (mouse sperm). Then, sperm were centrifuged at 325 RCF for 5–10 min and resuspended in the corresponding media. Sperm were allowed to attach to the recording chamber floor for 5 min. Mouse sperm were then overlaid with a coverslip coated with1 mg/ml Laminin Mouse Protein, Natural (catalog number 23017015, Gibco^®^), 0.1% (w/v) Poly-L-lysine in water (catalog number P8920-100ml, Sigma-Aldrich), or Cell-Tak (catalog number 354240, Corning Labware, Bedford, MA, United States). Laminin-coated cover slips were incubated at 4^*o*^C for at least 12 hrs before the experiment. Human sperm were overlaid with a coverslip coated with 0.1% Poly-L-lysine or 1 mg/ml Cell-Tak. Non-capacitated sperm were not exposed to HCO_3_^–^ at any time before the recordings. A perfusion device with an estimated exchange time of 10 s was used to apply test solutions. Sperm were pre-incubated with 15 mM (mouse) or 25 mM (human) HCO_3_^–^ for 20 min before recording. Then, HCO_3_^–^ was washed out for 2–4 min so that, at time “0” when the recording was started, no HCO_3_^–^ was present in the recording solution. PKA inhibitors and sAC inhibitor were added only during the pre-incubation period and were not present at the time of stimulation with KCl. Ionomycin (5 μM) and HS plus 2 mM CaCl_2_ were added at the end of the recordings as references of maximum fluorescence (F_*Iono*_).

Imaging was performed with a Leica DMi8 microscope equipped with a Zyla Andor sCMOS camera, and images were collected for 1.5 ms every 5–10 s over 10–30 min. Images were analyzed with Image J software. Fluorescence (F) of Fluo-4 changes was normalized to F_*Iono*_(*F*/*F_*iono*_)* after background subtraction. Calcium imaging data are presented as the average ± the standard deviation. The regions of interest were selected automatically and corroborated with ImageJ software. Pclamp 10 and Sigmaplot 12 were used to analyze data. Experiments were done at room temperature. Sperm in which the peak [Ca^2+^]_*i*_ reached 10% or more of the [Ca^2+^]_*i*_ in the presence of Ionomycin were considered to have responded to the stimuli.

### Intracellular pH Measurements

Sperm samples were incubated for 60 min in the presence or absence of the soluble adenylyl cyclase inhibitor TDI-10229 (donated by Lonnie Levin and Johen Buck’s labs) (Balbach et al., 2021) in capacitating TYH media or in non-capacitating HS media. After incubation, sperm pH was evaluated as previously described (Gunderson et al., 2021). Briefly, samples were incubated for 10 min with 0.5 μM BCECF-AM, washed, and resuspended in non-capacitating TYH media, media plus 15 mM bicarbonate, or media plus 15 mM HCO_3_^–^ and 5 μM TDI-10229. For each condition, high potassium-buffered solutions were used to calibrate the pH by adding 5 μM nigericin to equilibrate intracellular and extracellular pH and create a pH calibration curve. Fluorescence of BCECF was recorded as individual cellular events on a FACSCanto II TM cytometer (Becton Dickinson) (excitation 505 nm, emission 530 nm). Sperm intracellular pH was calculated by linearly interpolating the median of the histogram of BCECF fluorescence of the unknown sample to the calibration curve.

### Solutions

**Non-capacitating HS medium** (in mM): 135 NaCl, 5 KCl, 2 CaCl_2_, 1 MgSO_4_, 20 HEPES, 5 Glucose, 10 lactic acid (catalog number L13242, Alfa Aesar, Lancashire, United Kingdom), 1 sodium pyruvate (catalog number P2256, Sigma-Aldrich, Saint Louis, MO) at pH7.4 or as indicated. **Capacitated HS medium:** Same as non-capacitating HS medium plus 5 mg/ml bovine serum albumin (BSA, catalog number A2153, Sigma-Aldrich, Saint Louis, MO) and 15 mM NaHCO_3_ (catalog number S5761, Sigma-Aldrich) (Chavez et al., 2014). **50 mM KCl HS** (in mM): 90 NaCl, 50 KCl, 2 CaCl_2_, 1 MgSO_4_, 20 HEPES, 5 glucose, 10 lactic acid, 1 sodium pyruvate in pH as indicated. **Recording solution** (in mM): 110 NaCl, 4 KCl, 2 CaCl_2_, 1 MgCl_2_, 20 HEPES, 5 glucose, 10 lactic acid, 1 sodium pyruvate at pH as indicated. **KCl Recording solution** (in mM): 65 NaCl, 50 KCl, 2 CaCl_2_, 1 MgCl_2_, 20 HEPES, 5 glucose, 10 lactic acid, 1 sodium pyruvate at pH as indicated. **TYH medium (in mM)**:135 NaCl, 4.7 KCl, 1.7 CaCl2, 1.2 KH2PO4, 1.2 MgSO4, 5.6 glucose, 0.56 pyruvate, 10 HEPES, pH 7.4 adjusted at 37°C with NaOH.

pH of the solutions after addition of HCO_3_^–^ was measured with a Sartorious PB-10 basic bench top pH meter, with automatic temperature compensation.

### Other Reagents

Dimethyl sulfoxide (DMSO) (catalog number D8418, Sigma-Aldrich); CatSper inhibitor Mibefradil (catalog number 2198, Tocris Bioscience, Bristol, United Kingdom); PKI (Protein Kinase A inhibitor fragment 14-22, myristoylated trifluoroacetate salt, catalog number P9115; Sigma Aldrich); KT5720 (CAS number 108068-98-0, Cayman Chemicals).

## Results

### Mouse and Human Sperm at Similar Stages of Maturation Show Similar Increases in Intracellular Calcium Concentration ([Ca^2+^]_*i*_) in Response to Membrane Depolarization

**FIGURE 1:**
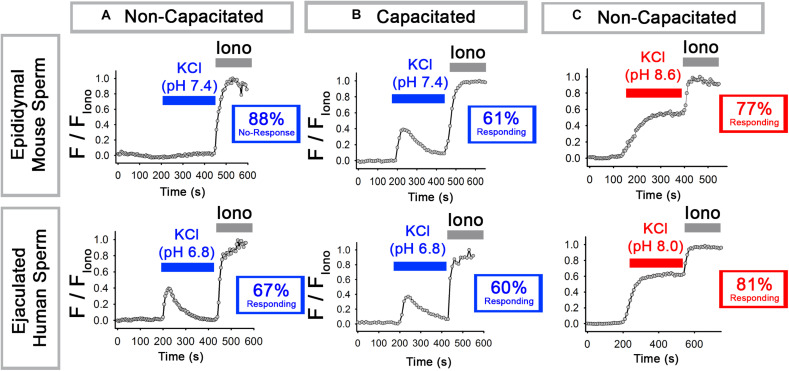
Epididymal mouse sperm and ejaculated human sperm show different [Ca^2+^]_*i*_ responses to KCl. **(A,B)** Representative traces of the most prevalent [Ca^2+^]_*i*_ responses to 50 mM KCl at (top) pH 7.4 in epididymal mouse sperm and (bottom) at pH 6.8 in ejaculated human sperm in **(A)** non-capacitating and **(B)** capacitating conditions. **(C)** Representative traces of the most prevalent [Ca^2+^]_*i*_ responses to 50 mM KCl at (top) pH 8.6 in epididymal mouse sperm and (bottom) at pH 8.0 ejaculated human sperm in non-capacitating conditions. Numbers in boxes indicate the percentage of sperm that did or did not respond (see Results and Methods for how “responding” was defined).

We first repeated previous experiments on mouse epidydimal sperm (Chavez et al., 2014) and confirmed that, at pH 7.4, KCl induced an increase in [Ca^2+^]_*i*_ in 12% of sperm in non-capacitating conditions and 61% of sperm in capacitating conditions (bicarbonate [HCO_3_^–^] plus bovine serum albumin). In this and all subsequent experiments, sperm in which the peak [Ca^2+^]_*i*_ reached 10% or more of the [Ca^2+^]_*i*_ in the presence of Ionomycin were considered to have responded. As expected, bypassing the increase in pH that occurs during capacitation by incubating the sperm at alkaline pH (pH 8.6) allowed 77% of the non-capacitated sperm to respond to KCl. The top row of Figure 1 shows representative traces of the most prevalent response in each condition.

We next performed similar experiments with human ejaculated sperm, which have been most commonly used to study sperm Ca^2+^ signaling and CatSper activation. For these experiments, we first tested pH 7.4 as in the mouse sperm experiments but found that it caused Ca^2+^ responses with varying kinetics (not shown). Thus, we instead used pH 6.8 and pH 8.0 because they yielded consistent and reproducible Ca^2+^ responses similar to those obtained with mouse sperm at pH 7.4 and pH 8.6. In ejaculated human sperm at pH 6.8, membrane depolarization with KCl triggered an increase in [Ca^2+^]_*i*_ in 67% of sperm in non-capacitating conditions and 60% of sperm in capacitating conditions. As with mouse epididymal sperm, bypassing the increase in pH that occurs during capacitation with alkaline pH (pH 8.0) allowed a high percentage (81%) of sperm to respond to KCl. The bottom row of Figure 1 shows representative traces of the most prevalent response in each condition. We conclude that, at low pH in non-capacitating conditions, epididymal mouse and ejaculated human sperm sharply differ in their abilities to increase [Ca^2+^]_*i*_ in response to KCl-mediated depolarization.

To determine whether sperm from the two species at similar stages of maturation would respond to KCl-induced membrane depolarization similarly, we compared epididymal mouse sperm to epididymal human sperm and compared ejaculated mouse sperm to ejaculated human sperm. In each case, we assessed response by measuring the percentage of sperm that responded to the KCl stimulus. Additionally, given that the slope of the [Ca^2+^]_*i*_ increase is a good indicator of the number of CatSper channels that are open in response to membrane depolarization (Wennemuth et al., 2003), we compared the rate of [Ca^2+^]_*i*_ increase in the responsive sperm during the first 20 s of the depolarization-evoked response.

**FIGURE 2:**
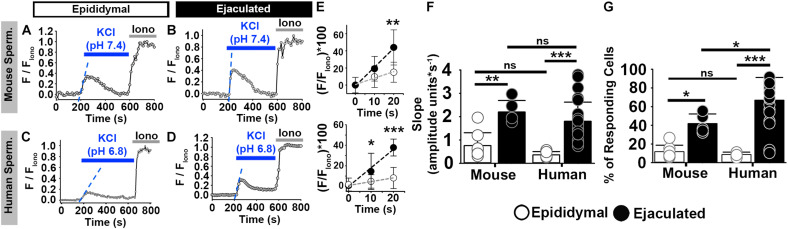
Mouse and human sperm at similar stages of maturation show similar increases in [Ca^2+^]_*i*_ in response to KCl. **(A–D)** [Ca^2+^]_*i*_ traces from the epididymal and ejaculated sperm responsive to KCl in non-capacitated conditions. **(E)** Linear fit of the averaged normalized fluorescence in the first 20 s after KCl addition [blue dashed lines in panels **(A–D)**] in responding sperm. **(F)** Graph of the slope values of the best-fit regression lines. Mean values were as follows: epididymal mouse sperm, 0.76 ± 0.547 amplitude units * s^–1^ (8 mice, 307 sperm); ejaculated mouse sperm, 2.20 ± 0.494 amplitude units *s^–1^ (5 mice, 170 sperm); epididymal human sperm, 0.36 ± 0.137 amplitude units *s^–1^ (5 samples, 191 sperm); and ejaculated human sperm, 1.80% ± 0.82 amplitude units *s^–1^ (26 samples, 1836 sperm). **(G)** Graph of the percentage of responsive sperm. Mean values were as follows: epididymal mouse sperm, 11.80% ± 6.9 (8 mice, 307 sperm); ejaculated mouse sperm, 41.9% ± 10.2 (5 mice, 170 sperm); epididymal human sperm, 8.72% ± 2.54 (5 samples, 191 sperm); and ejaculated human sperm, 66.94% ± 24.30 (26 samples, 1836 sperm). *P < 0.050, **P < 0.010, ***P < 0.001, and ns, non-significant by one-way ANOVA. Error bars indicate standard deviation. Iono = 5 μM Ionomycin + 2 mM Ca^2+^.

In non-capacitating conditions, 12% of mouse epididymal sperm increased [Ca^2+^]_*i*_ in response to high external KCl at pH 7.4 (Figures 2A,G). Similarly, 8% of human epididymal sperm increased [Ca^2+^]_*i*_ in response to high external KCl pH 6.8 (Figures 2C,G). In contrast, 42% of mouse ejaculated sperm and 67% of human ejaculated sperm increased [Ca^2+^]_*i*_ in response to high external KCl (Figures 2B,D,G). In both mouse and human sperm, a significantly higher percentage of ejaculated sperm responded than did epididymal sperm (Figure 2G). When we examined only those sperm in each condition that responded, we found that the rate of [Ca^2+^]_*i*_ increase was significantly higher in ejaculated mouse and human sperm than in epididymal mouse and human sperm, respectively (Figures 2E,F).

In capacitating conditions, mouse epididymal sperm showed [Ca^2+^]_*i*_ increases in response to high external KCl at low pH, and both non-capacitated and capacitated sperm from human and mouse species showed [Ca^2+^]_*i*_ increases in response to high external KCl at high pH (Supplementary Figure 1; Wennemuth et al., 2000; Chavez et al., 2014). As expected (Xia et al., 2007; Chavez et al., 2014), capacitated epididymal sperm from CatSper knockout mice did not show [Ca^2+^]_*i*_ increases in response to high external KCl (Supplementary Figure 1C). Similarly, in ejaculated human sperm, the [Ca^2+^]_*i*_ increase in response to high external KCl was inhibited by the CatSper channel blocker Mibefradil (Supplementary Figure 1F). Together, these results indicate that human and mouse ejaculated sperm show comparable [Ca^2+^]_*i*_ increases in response to membrane depolarization by high extracellular KCl. This depolarization similarly activates CatSper channels in mouse and human sperm at similar maturational stages.

### Bicarbonate (HCO_3_^–^) Increases [Ca^2+^]_*i*_ Responses to Neutral and Alkaline Depolarization in Both Mouse and Human Epididymal Sperm

The main difference between epididymal and ejaculated sperm is that ejaculated sperm are exposed to semen. Thus, we aimed to identify the component in semen that was responsible for the differences in [Ca^2+^]_*i*_ increases in response to membrane depolarization between epididymal and ejaculated sperm. We focused on bicarbonate (HCO_3_^–^) because it is a major component of semen (Okamura et al., 1985) and influences sperm function and intracellular Ca^2+^ regulation (Austin, 1952; Austin and Bishop, 1958; Wennemuth et al., 2003; Chavez et al., 2014). For example, Wennemuth et al. showed that a pulse of HCO_3_^–^ facilitated Ca^2+^ increase in mouse sperm by enhancing the rate of depolarization-evoked [Ca^2+^]_*i*_ increase (Wennemuth et al., 2003). Additionally, de la Vega-Beltran et al. (2012) and Chavez et al. (2014) showed that exposure to HCO_3_^–^ and bovine serum albumin triggered Ca^2+^ responses in mouse sperm, and Orta et al. (2018) showed that CatSper activity was modulated by HCO_3_^–^ in mouse sperm.

**FIGURE 3:**
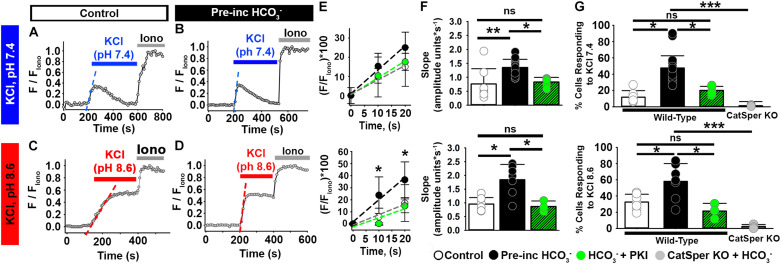
HCO_3_^–^ enhances the depolarization-evoked responses from mouse epididymal NC sperm and its effect is inhibited by the PKA inhibitor PKI. **(A–D)** Representative traces of [Ca^2+^]_*i*_ responses to 50 mM KCl at **(A,B)** pH 7.4 and **(C,D)** pH 8.6 in mouse epididymal sperm in non-capacitating conditions. **(E)** Linear fit of the averaged normalized fluorescence in the first 20 s after KCl addition [blue or red dashed lines in panels **(A–D)**] in responding sperm. **(F)** Graph of the slope values of the best-fit regression line for each condition. Mean values at pH 7.4 were as follows: control, 0.76 ± 0.547 amplitude units * s^–1^ (8 mice, 307 sperm); HCO_3_^–^, 1.35 ± 0.297 amplitude units *s^–1^ (15 mice, 1035 sperm); and HCO_3_^–^ + PKI, 0.83 ± 0.166 amplitude units *s^–1^ (5 mice, 264 sperm). Mean values at pH 8.6 were as follows: control, 0.95 ± 0.237 amplitude units * s^–1^ (7 mice, 243 sperm); HCO_3_^–^, 1.84 ± 0.556 amplitude units *s^–1^ (7 mice, 281 sperm); and HCO_3_^–^ + PKI, 0.86 ± 0.203 amplitude units *s^–1^ (3 mice, 126 sperm). **(G)** Graph of the percentage of responsive sperm. Mean values at pH 7.4 were as follows: control, 11.80% ± 6.9 (8 mice, 307 sperm); HCO_3_^–^, 47.6% ± 19.39 (15 mice, 1035 sperm); HCO_3_^–^ + PKI, 20.1% ± 5.30 (5 mice, 264 sperm); and HCO_3_^–^ in CatSper KO mice, 1.37% ± 0.623 (5 mice, 137 sperm). Mean values at pH 8.6 were as follows: control, 32.6% ± 9.58 (7 mice, 243 sperm); HCO_3_^–^, 58.0% ± 21.940 (7 mice, 281 sperm); HCO_3_^–^ + PKI, 21.45% ± 9.513 (3 mice, 126 sperm); and HCO_3_^–^ in CatSper KO mice, 2.34% ± 2.524 (3 mice, 95 sperm). *P < 0.050, **P < 0.010, ***P < 0.001, and ns, non-significant by one-way ANOVA. Error bars indicate standard deviation. Iono = 5 μM Ionomycin + 2 mM Ca^2+^.

To assess the effect of HCO_3_^–^, we pre-incubated mouse epididymal sperm without or with 15 mM HCO_3_^–^ (a concentration similar to that in seminal fluid) (Okamura et al., 1985) for 20 min. Whereas 12% of sperm not exposed to HCO_3_^–^ showed increased [Ca^2+^]_*i*_ in response to high extracellular KCl at pH 7.4 (Figures 3A,G), 48% of sperm exposed to HCO_3_^–^ showed increased [Ca^2+^]_*i*_ in response to high extracellular KCl at pH 7.4 (Figures 3B,G). Exposure to HCO_3_^–^ also increased the percentage of cells showing increased [Ca^2+^]_*i*_ in response to high extracellular KCl at pH 8.6 (Figures 3C,D,G). When we only examined the responding sperm in each condition, we found that pre-incubation with HCO_3_^–^ enhanced the rate of depolarization-evoked [Ca^2+^]_*i*_ increase in mouse epididymal sperm (Figures 3E,F), indicating an increase in the number of open CatSper channels in response to KCl depolarization. These [Ca^2+^]_*i*_ increases were not observed in sperm from CatSper knock-out mice (Figure 3G). Importantly, 15 mM HCO_3_^–^ did not significantly increase the pH of the HS media or the recording solution (Supplementary Figure 2). Moreover, HCO_3_^–^ was not present in the chamber during the recordings.

**FIGURE 4:**
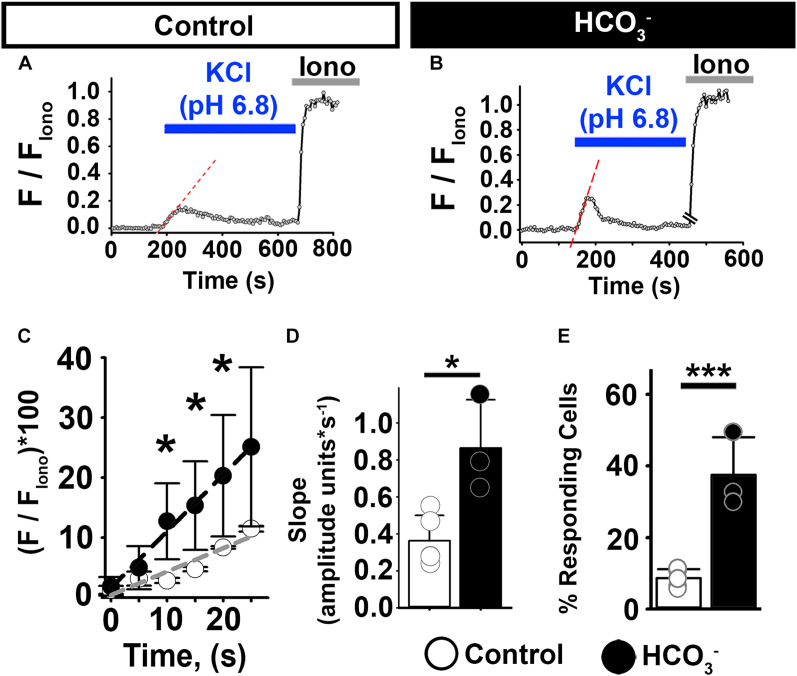
HCO_3_^–^ enhances [Ca^2+^]_*i*_ responses to KCl in human epididymal sperm. **(A,B)** Representative traces of [Ca^2+^]_*i*_ responses to 50 mM KCl at pH 6.8 in epididymal human sperm in non-capacitating conditions **(A)** without and **(B)** after pre-incubation with HCO_3_^–^. **(C)** Linear fit of the averaged normalized fluorescence in the first 20 s after KCl addition. **(D)** Graph of slope values of the best-fit regression line for each condition. Mean values were as follows: control, 0.36 ± 0.137 amplitude units * s^–1^ (4 samples, 62 sperm); and HCO_3_^–^, 0.86 ± 0.260 amplitude units *s^–1^ (3 samples, 52 sperm). **(E)** Graph of the percentage of responsive sperm. Mean values were as follows: control, 8.72% ± 2.54 (4samples, 62 sperm); and pre-incubation with HCO_3_^–^, 37.5% ± 10.54 (3 samples, 52 sperm). *P < 0.050, ***P < 0.001 by independent t-test. Error bars indicate standard deviation. Iono = 5 μM Ionomycin + 2 mM Ca^2+^.

To assess the effect of HCO_3_^–^ on [Ca^2+^]_*i*_ responses in non-capacitated human epididymal sperm, we incubated them in the presence or absence of HCO_3_^–^ for 20–30 min and then exposed them to high external KCl. High external KCl at pH 6.8 induced [Ca^2+^]_*i*_ increases in 9% of the human epididymal sperm in control conditions (Figures 4A,E). In contrast, high external KCl at pH 6.8 induced [Ca^2+^]_*i*_ increases in 37% of sperm pre-incubated with HCO_3_^–^ (Figures 4B,E). Pre-incubation with HCO_3_^–^ also significantly increased the rate of depolarization-evoked [Ca^2+^]_*i*_ increase in human epididymal sperm (Figures 4C,D), indicating an increase in the number of open CatSper channels in response to KCl depolarization. These results indicate that [Ca^2+^]_*i*_ responses to membrane depolarization depend on HCO_3_^–^ in both mouse and human sperm.

### HCO_3_^–^ Activates the cAMP/PKA Pathway and Sensitizes CatSper to Membrane Depolarization in Sperm From Both Mice and Humans

One of the early events of sperm capacitation, which can occur as soon as sperm are ejaculated, is HCO_3_^–^-induced activation of soluble adenylyl cyclase (sAC), leading to increased intracellular cyclic adenosine monophosphate (cAMP) and activation of protein kinase A (PKA) (Chen et al., 2000; Hess et al., 2005; Xie et al., 2006; Visconti, 2009). Given a report suggesting that CatSper channels are modulated by PKA (Orta et al., 2018), we wondered whether the effect of HCO_3_^–^ on the Ca^2+^ responses in epididymal sperm depended on PKA activation. Recent work from Orta et al. showed that the PKA inhibitor PKI reduced HCO_3_^–^-induced [Ca^2+^]_*i*_ increases in mouse epididymal sperm without directly blocking mouse CatSper currents (Orta et al., 2018). Thus, we incubated mouse epididymal sperm in HCO_3_^–^ in the absence or presence of 10 μM PKI for 20 min before recording, then stimulated them with KCl in the absence of HCO_3_^–^ or PKI. Compared to sperm not pre-treated with PKI, significantly fewer sperm pre-treated with PKI showed an increase in [Ca^2+^]_*i*_ in response to high external KCl at both pH 7.4 and pH 8.6 (Figure 3G). The rate of [Ca^2+^]_*i*_ increase of the responsive cells was also significantly lower in cells pre-incubated with HCO_3_^–^ in the presence than in the absence of PKI (Figures 3E,F).

**FIGURE 5:**
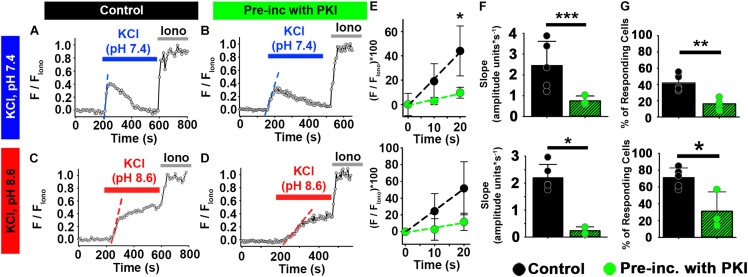
PKA inhibition impairs the effect of HCO_3_^–^ on depolarization-evoked Ca^2+^ responses in ejaculated mouse sperm. **(A–D)** Representative traces of [Ca^2+^]_*i*_ responses to 50 mM KCl at pH 7.4 and pH 8.6 in ejaculated mouse sperm in **(A,C)** control conditions and **(B,D)** after pre-incubation with 10 μM PKI. **(E)** Linear fit of the averaged normalized fluorescence in the first 20 s after KCl addition. **(F)** Graph of slope values of the best-fit regression line for each condition. Mean values at pH 7.4 were as follows: control, 2.20 ± 0.494 amplitude units * s^–1^ (5 mice, 137 sperm); and PKI pre-incubation, 0.237 ± 0.144 amplitude units *s^–1^ (4 mice, 86 sperm). Mean values at pH 8.6 were as follows: control, 2.45 ± 1.161 amplitude units * s^–1^ (5 mice, 107 sperm); and PKI pre-incubation, 0.753 ± 0.232 amplitude units *s^–1^ (3 mice, 81 sperm). **(G)** Graph of the percentage of responsive sperm. Mean values at pH 7.4 were as follows: control, 41.92% ± 10.22 (5 mice, 137 sperm); and PKI pre-incubation, 16.30% ± 7.01 (4 mice, 82 sperm). Mean values at pH 8.6 were as follows: control, 70.96% ± 11.50 (5 mice, 107 sperm); and PKI pre-incubation, 31.22% ± 22.80 (3 mice, 81 sperm). *P < 0.050, **P < 0.010, and ***P < 0.001 by independent *t*-test. Error bars indicate standard deviation. Iono = 5 μM Ionomycin + 2 mM Ca^2+^.

We next asked whether the cAMP/PKA pathway was also involved in [Ca^2+^]_*i*_ increases induced by external KCl in ejaculated mouse sperm. At pH 7.4 (Figures 5A,B), treatment with PKI reduced the percentage of ejaculated mouse sperm that showed increased [Ca^2+^]_*i*_ in response to high extracellular KCl from 42 to 16% (Figure 5G, top panel). PKI also significantly reduced the rate of [Ca^2+^]_*i*_ increase in the responsive sperm (Figures 5E,F). Likewise, at pH 8.6 (Figures 5C,D), treatment with PKI reduced the percentage of ejaculated sperm that showed increased [Ca^2+^]_*i*_ in response to high extracellular KCl from 71 to 32% (Figure 5G, bottom panel). PKI also significantly reduced the rate of [Ca^2+^]_*i*_ increase in the responsive sperm (Figures 5E,F). These results indicated that PKI reduced the number of CatSper channels that opened in response to membrane depolarization in mouse ejaculated sperm.

**FIGURE 6:**
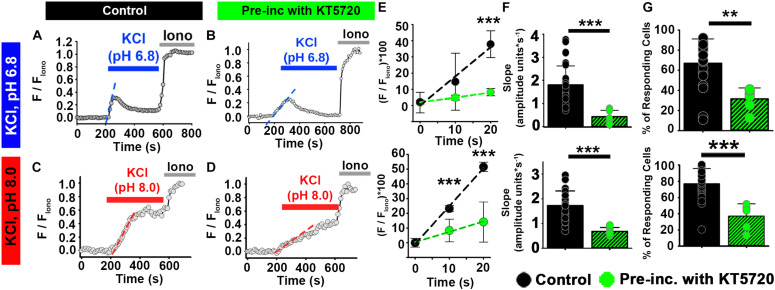
PKA inhibitor KT5720 impairs the effect of HCO_3_^–^ on depolarization-evoked Ca^2+^ responses in ejaculated human sperm. **(A–D)** Representative traces of [Ca^2+^]_*i*_ responses to 50 mM KCl at **(A,B)** pH 6.8 and **(C,D)** pH 8.0 in ejaculated human sperm in non-capacitating conditions in **(A,C)** control and **(B,D)** after pre-incubation with KT5720. **(E)** Linear fit of the averaged normalized fluorescence in the first 20 s after KCl addition. **(F)** Graph of slope values of the best-fit regression line for each condition. Mean values at pH 6.8 were as follows: control, 1.80 ± 0.82 amplitude units * s^–1^ (26 samples, 1836 sperm); and KT5720 pre-incubation, 0.426 ± 0.277 amplitude units *s^–1^ (6 samples, 624 sperm). Mean values at pH 8.0 were as follows: control, 1.73 ± 0.594 amplitude units * s^–1^ (26 samples, 3036 sperm); and KT5720 pre-incubation, 0.678 ± 0.162 amplitude units *s^–1^ (5 samples, 974 sperm). **(G)** Graph of the percentage of responsive sperm. Mean values at pH 6.8 were as follows: control, 66.94% ± 24.30 (26 samples, 1836 sperm); and KT5720 pre-incubation, 31.57% ± 10.87 (6 samples, 624 sperm). Mean values at pH 8.6 were as follows: control, 77.04% ± 18.67 (26 samples, 3036 sperm); and KT5720 pre-incubation, 36.90% ± - 15.33 (6 samples, 974 sperm). **P < 0.010, ***P < 0.001 by independent *t*-test. Error bars indicate standard deviation. Iono = 5 μM Ionomycin + 2 mM Ca^2+^.

We wanted to determine whether PKA was required for the [Ca^2+^]_*i*_ increase in ejaculated human sperm. However, because PKI has been reported to directly inhibit human CatSper currents (47), we instead used KT5720, a PKA inhibitor that does not directly inhibit human CatSper currents (Wang et al., 2020). Pre-incubation of ejaculated human sperm with HCO_3_^–^ in the presence of 50 μM KT5720 reduced the percentage of sperm responding from 67 to 32% (Figures 6A–D,G). Additionally, KT5720 significantly decreased the rate of [Ca^2+^]_*i*_ increase in responsive sperm (Figures 6E,F). These results indicate that the cAMP/PKA pathway is involved in the [Ca^2+^]_*i*_ responses to membrane depolarization in ejaculated sperm from both mice and humans.

### Activation of Soluble Adenylyl Cyclase Is Involved in [Ca^2+^]_*i*_ Increases Induced by Bicarbonate

**FIGURE 7:**
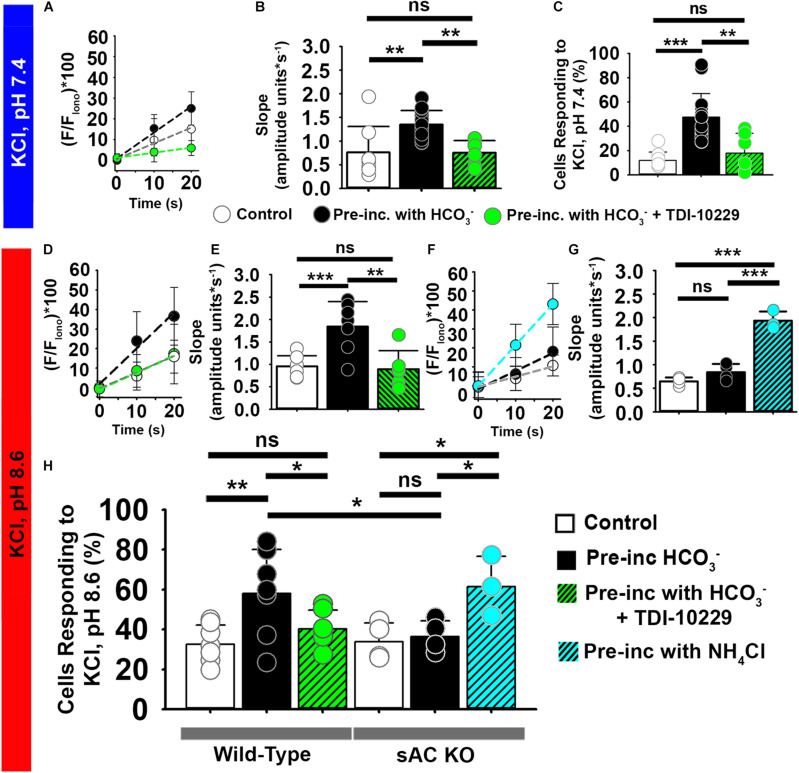
The soluble adenylyl cyclase (sAC) is required for [Ca^2+^]_*i*_ increases evoked by KCl after HCO_3_^–^ sensitization in epididymal mouse sperm. **(A,D,F)** Linear fit of the averaged normalized fluorescence in the first 20 s after KCl addition at **(A)** pH 7.4 and **(D,F)** pH 8.6. Data were obtained from epididymal **(A,D)** wild-type mouse sperm and from **(G)** sAC KO mouse sperm in control conditions (white) and after pre-incubation with HCO_3_^–^ (black), HCO_3_^–^ + PKA inhibitor TDI-10229 (Green), or NH_4_Cl (Blue). **(B)** Graph of slope values of the best-fit regression line for each condition. Mean values at pH 7.4 were as follows: control, 0.76 ± 0.547 amplitude units * s^–1^ (8 mice, 307 sperm); incubation with HCO_3_^–^, 1.35 ± 0.297 amplitude units *s^–1^ (15 mice, 1035 sperm); and incubation with TDI-10229, 0.75 ± 0.259 amplitude units *s^–1^ (6 mice, 364 sperm). **(C)** Graph of the percentage of responsive sperm. Mean values at pH 7.4 were as follows: control, 11.80% ± 6.9 (8 mice, 307 sperm); HCO_3_^–^, 47.6% ± 19.39 (15 mice, 1035 sperm); and HCO_3_^–^ + TDI-10229,17.8% ± 16.33 (6 mice, 364 sperm). **(E)** Slope values per animal of the best-fit linear regression of the rise in KCl at pH 8.6 in different conditions. Mean values at pH 8.6 were as follows: control, 0.95 ± 0.237 amplitude units * s^–1^ (7 mice, 243 sperm); HCO_3_^–^, 1.84 ± 0.556 amplitude units *s^–1^ (7 mice, 281 sperm); and HCO_3_^–^ + TDI-10229, 0.88 ± 0.413 amplitude units *s^–1^ (6 mice, 126 sperm). **(G)** Graph of slope values of the best-fit regression line for each condition in the sAC KO mice. Mean values at pH 8.6 were as follows: control, 0.645 ± 0.084 amplitude units * s^–1^ (4 mice, 123 sperm); HCO_3_^–^, 0.840 ± 0.173 amplitude units *s^–1^ (4 mice, 109 sperm); and NH_4_Cl, 1.932 ± 0.197 amplitude units *s^–1^ (3 mice, 74 sperm). **(H)** Graph of the percentage of responsive sperm. Mean values for wild-type at pH 8.6 were as follows: control, 32.62% ± 9.58 (7 mice, 243 sperm); HCO_3_^–^, 58.05% ± 21.94 (7 mice, 281 sperm); and HCO_3_^–^ + TDI-10229, 40.25% ± 9.51 (6 mice, 126 sperm). Mean values for sAC knockout at pH 8.6 were as follows: control, 33.80% ± 9.44 (4 mice, 91 sperm); HCO_3_^–^, 36.36% ± 7.96 (4 mice, 83 sperm); and NH_4_Cl, 61.47% ± 15.20 (3 mice, 74 sperm). *P < 0.050, **P < 0.010, ***P < 0.001, and ns, non-significant by one-way ANOVA. Error bars indicate standard deviation. Iono = 5 μM Ionomycin + 2 mM Ca^2+^.

As mentioned above, HCO_3_^–^ regulates the production of cAMP and PKA activity through stimulation of a soluble adenylyl cyclase (sAC) (Chen et al., 2000; Hess et al., 2005; Xie et al., 2006; Visconti, 2009). Moreover, KCl-induced Ca^2+^ entry is impaired in sAC (*Adcy10*) knock-out mice (Xie et al., 2006). To determine whether sAC was required for the depolarization-induced [Ca^2+^]_*i*_ increase in sperm, we took two approaches. First, in a genetic approach, we isolated epididymal sperm from sAC knockout mice. HCO_3_^–^ had no effect on the percentages of sperm from sAC knockout mice that increased [Ca^2+^]_*i*_ in response to extracellular KCl (Figure 7H). Likewise, HCO_3_^–^ had no effect on the rate of [Ca^2+^]_*i*_ increase in responding sperm from sAC knockout mice (Figures 7F,G).

**FIGURE 8:**
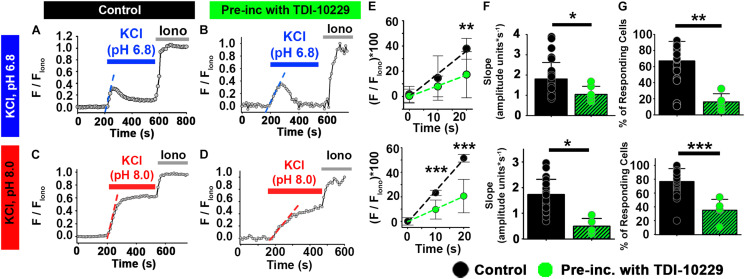
The sACspecific inhibitor TDI-10229 impairs [Ca^2+^]i responses to KCl in ejaculated human sperm. **(A–D)** Representative traces of [Ca^2+^]_*i*_ responses to 50 mM KCl at **(A,B)** pH 6.8 and **(C,D)** pH 8.0 in ejaculated human sperm in non-capacitating conditions in **(A,C)** control and **(B,D)** after pre-incubation with TDI-10229. **(E)** Linear fit of the averaged normalized fluorescence in the first 20 s after KCl addition at pH 6.8 and pH 8.0. **(F)** Graph of slope values of the best-fit regression line for each condition. Mean values at pH 6.8 were as follows: control, 1.80 ± 0.82 amplitude units * s^–1^ (26 samples, 1836 sperm); and TDI-10229 incubation, 0.426 ± 0.276 amplitude units *s^–1^ (5 samples, 2279 sperm). Mean values at pH 8.0 were as follows: control, 1.73 ± 0.594 amplitude units * s^–1^ (26 samples, 3036 sperm); and TDI-10229 incubation, 0.491 ± 0.305 amplitude units *s^–1^ (5 samples, 2279 sperm). **(G)** Graph of the percentage of responsive sperm. Mean values at pH 6.8 were as follows: control, 66.94% ± 24.30 (26 samples, 1836 sperm); and TDI-10229 pre-incubation, 16.18% ± 10.10 (5 samples, 2279 sperm). Mean values at pH 8.0 were as follows: control, 77.04% ± 18.67 (26 samples, 3036 sperm); and TDI-10229 pre-incubation, 35.43% ± 15.77 (5 samples, 2279 sperm). *P < 0.050, **P < 0.010, and ***P < 0.001 by independent *t*-test. Error bars indicate standard deviation. Iono = 5 μM Ionomycin + 2 mM Ca^2+^.

Second, in a pharmacologic approach, we treated epididymal sperm from wild-type mice with the sAC-specific inhibitor TDI-10229 (Balbach et al., 2021) and found that it prevented an increase in the percentage of sperm responding to high extracellular KCl (Figures 7C,H). Treatment with TDI-10229 also reduced the rate of [Ca^2+^]_*i*_ increase in responsive sperm (Figures 7A,B,D,E). Likewise, TDI-10229 prevented the increase in the percentage of ejaculated human sperm responding to high extracellular KCl (Figure 8G) and reduced the rate of [Ca^2+^]_*i*_ increase in the responsive sperm (Figures 8A–F). Together, these data indicate that activation of CatSper channels by membrane depolarization is facilitated by activation of the HCO_3_^–^-sAC-cAMP/PKA pathway in both mouse and human sperm.

### The Effect of HCO_3_^–^ Is Mediated by an Increase in Intracellular pH Induced by sAC Activation

**FIGURE 9:**
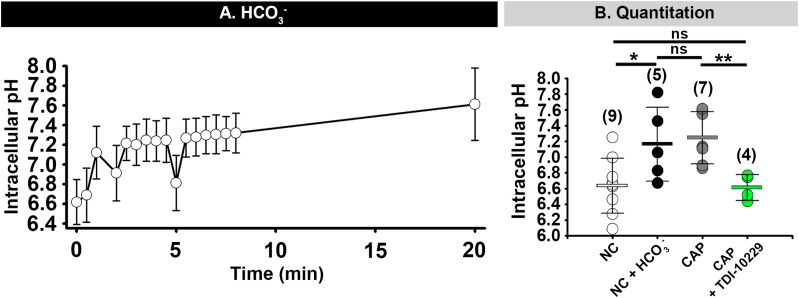
HCO_3_^–^- induced increase of intracellular pH depends on sAC activity. **(A)** Mean and standard deviation values of intracellular pH in mouse epididymal sperm after addition of 15 mM HCO_3_^–^ at 0 min) (*n* = 3 mice). **(B)** Graph showing sperm intracellular pH values after 60 min incubation in the indicated conditions. NC, non-capacitating; CAP, capacitating. Horizontal bars indicate mean and standard deviation. Mean values for intracellular pH were as follows: NC, 6.64 ± 0.349 (9 mice); NC + HCO_3_^–^, 7.17 ± 0.469 (5 mice); CAP, 7.25 ± 0.331 (7 mice); and CAP + TDI10229, 6.62 ± 0.164 (4 mice) respectively. Numbers in parentheses are numbers of animals. *P < 0.05 and **P < 0.01 by one-way ANOVA. ns, non-significant.

Several studies have revealed that HCO_3_^–^ raises sperm intracellular pH (pH_*i*_) (Demarco et al., 2003; Chavez et al., 2019; Matamoros-Volante and Trevino, 2020) and that HCO_3_^–^ can activate CatSper channels in mouse sperm (Orta et al., 2018). Therefore, we investigated whether the [Ca^2+^]_*i*_ changes induced by HCO_3_^–^ were due to a change in pH_*i*_. We found that addition of HCO_3_^–^ to the media (which only raised the media pH by 0.1 unit; Supplementary Figure 2) caused a 0.4–0.5 unit increase in non-capacitated mouse epididymal sperm pH_*i*_ within 1–2 min (Figure 9A). This increase in pH_*i*_ was similar to the increase in pH_*i*_ seen in capacitated mouse sperm and was completely inhibited by incubation with the sAC inhibitor TDI-10229 (Figure 9B). Finally, we asked whether we could bypass the need for sAC by treating sperm with 20 mM NH_4_Cl, which increases pH_*i*_. This treatment increased the percent of sperm from sAC knock-out mice that increased [Ca^2+^]_*i*_ in response to high extracellular KCl from 33.8 to 61.5% (Figure 7H). These data indicate that the change in pH_*i*_ induced by sAC/PKA activation is responsible for sensitizing CatSper channels to membrane depolarization.

## Discussion

**FIGURE 10:**
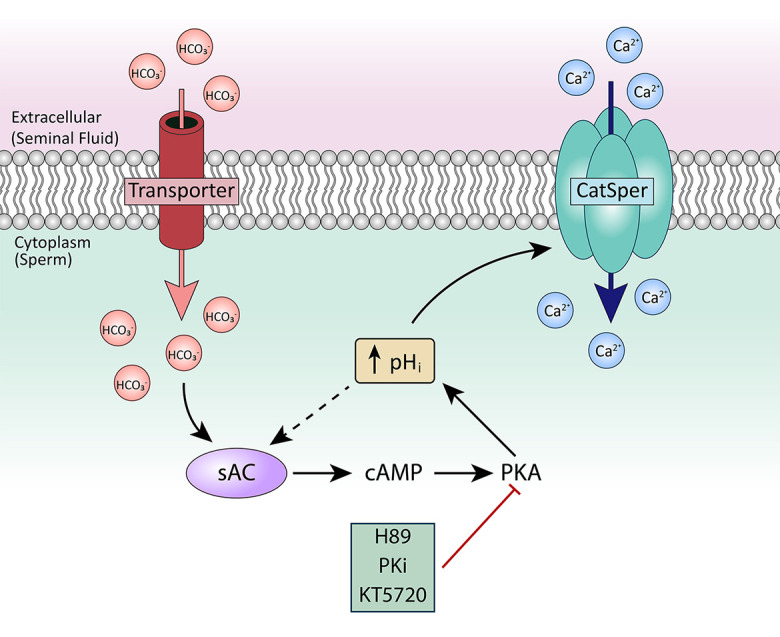
Proposed model for CatSper activation in ejaculated human and mouse sperm. After ejaculation, sperm are exposed to HCO_3_^–^ in the seminal fluid. HCO_3_^–^ activates sAC, leading to production of cAMP, which activates PKA. PKA stimulates alkalinization of the cytoplasm, which sensitizes CatSper to respond to membrane depolarization.

Together, our data indicate that the model shown in Figure 10 is responsible for CatSper sensitization to membrane depolarization in both mouse and human sperm. First, ejaculated sperm from both mice and humans showed substantial [Ca^2+^]_*i*_ increases in response to high external KCl at pH 7.4 and pH 6.8, respectively. The greater [Ca^2+^]_*i*_ responses in ejaculated sperm than in epididymal sperm were reflected both by increases in the percentage of responsive sperm and by faster rates of [Ca^2+^]_*i*_ increase in the responsive sperm. Second, although [Ca^2+^]_*i*_ did not increase in response to high external KCl at low pH in either mouse epididymal or human epididymal sperm, [Ca^2+^]_*i*_ increases occurred in mouse epididymal and human epididymal sperm exposed to HCO_3_^–^. This result indicates that exposure to semen, which contains HCO_3_^–^, is important for CatSper activation. Third, PKA inhibitors prevented the increases in [Ca^2+^]_*i*_ in response to high external KCl in both mouse and human ejaculated sperm and mouse epididymal sperm pre-incubated with HCO_3_^–^. These PKA inhibitors reduced both the percentage of responsive sperm and the rate of [Ca^2+^]_*i*_ increase in the responsive sperm. Finally, both genetic ablation of sAC and its pharmacological inhibition prevented the [Ca^2+^]_*i*_ increases induced by HCO_3_^–^. Thus, our data reveal that CatSper is activated by KCl-induced membrane depolarization similarly in mouse and human sperm at similar stages. Moreover, our data suggest that regulation of CatSper activation by cAMP/PKA starts when the sperm cells encounter the seminal fluid.

Our data help resolve a question in the field regarding CatSper. Although CatSper is known to be the principal Ca^2+^ channel controlling sperm motility in numerous species (Lishko and Mannowetz, 2018), whether the mechanisms of activation are conserved has been unclear. For example, in sea urchin, mouse, and human sperm, CatSper channels are activated by membrane depolarization and by increases in intracellular pH (Kirichok et al., 2006; Seifert et al., 2015; Hwang et al., 2019), suggesting conserved mechanisms. Conversely, human CatSper activity is modulated by nanomolar concentrations of progesterone and prostaglandins both in epididymal and ejaculated samples (Lishko et al., 2011; Strunker et al., 2011), whereas mouse CatSper seems not to be modulated by these hormones (Lishko et al., 2011). Previous work from many labs has shown that HCO_3_^–^ modulates Ca^2+^ entry in mouse and human sperm (Wennemuth et al., 2003; Bedu-Addo et al., 2005; Chavez et al., 2014; Luque et al., 2018). Recently, mouse CatSper was proposed to be activated by HCO_3_^–^ through activation of sAC and PKA (Orta et al., 2018), whereas this mechanism was thought not to apply to human CatSper (Wang et al., 2020). Our work resolves this issue by comparing CatSper activation in mouse and human sperm before and after ejaculation. Thus, our data illustrate the importance of comparing sperm from similar stages of maturation and after similar environmental exposures.

In contrast with our results, Wang et al. (2020) recently reported that CatSper was not modulated by PKA. Several differences in the approach and interpretation of data could explain the different outcomes. First, most of the experiments done by Wang et al. used progesterone to induce Ca^2+^ responses in human sperm. We did not examine the effect of HCO_3_^–^ on the progesterone response, which might differ from the effect of HCO_3_^–^ on the KCl depolarization response. In the few experiments done by Wang et al. using KCl at pH 8.6, they measured the amplitude of [Ca^2+^]_*i*_ and not the rate of [Ca^2+^]_*i*_ increase. We argue that the rate of [Ca^2+^]_*i*_ increase is a better indicator of the effect of HCO_3_^–^ on the depolarizing-evoked Ca^2+^ responses than the absolute amplitude. The amplitude of the response might be affected by Ca^2+^ extrusion and intracellular Ca^2+^ buffers, which can vary upon changes in membrane potential, pH_*i*_, or both.

Second, Wang et al. suggested that the effect of HCO_3_^–^ on CatSper activation could be non-specific and due to HCO_3_^–^-induced changes in the pH of the media and the cytosol (Wang et al., 2020). Specifically, the authors showed that addition of 50 mM HCO_3_^–^ significantly changed the pH of the recording solutions over 120 min, and these changes in pH of the media could change the sperm pH_*i*_. We performed similar experiments measuring the pH of all of our solutions 30 min (the timeline of our experiments) after adding 15, 25, or 50 mM HCO_3_^–^. Although 50 mM HCO_3_^–^ substantially increased the pH, 15 mM HCO_3_^–^ only increased the pH of the solutions by 0.05–0.1 units after 30 min (Supplementary Figure 2). In addition, we washed HCO_3_^–^ out during stimulation with KCl. We also showed that, in non-capacitated sperm, the pH_*i*_ increased by 0.5–0.8 units after 10 min incubation in 15 mM HCO_3_^–^. Thus, HCO_3_^–^ caused the sperm pH_*i*_ to increase 5–10-fold more than the media pH. Thus, any changes to external pH were not likely to be responsible for the pH_*i*_ changes of 0.5–0.6 units we observed after applying HCO_3_^–^.

Third, we used different experimental systems than Wang et al. Most notably, Wang et al. performed experiments on sperm populations with a multi-well plate reader (Fluostar Omega, BMG Labtech) in which solutions cannot be changed. Concentrations of ions and drugs can be modified only by adding concentrated solutions such as 50 mM HCO_3_^–^ and 98.5 mM KCl to yield final concentrations of approximately 25 mM HCO_3_^–^ and 50 mM KCl. Given the diffusion of the ions in the first minute of the recordings, a considerable number of cells could have been exposed to the higher concentrations of HCO_3_^–^ and KCl. Therefore, the effects on Ca^2+^ signals reported at 25 mM HCO_3_^–^ and KCl at pH 8.6 could be underestimated, as many cells could have been exposed to a saturating stimulus of 98.5 mM KCl and 50 mM HCO_3_^–^ in the controls at the moment of injection. In contrast, we measured [Ca^2+^]_*i*_ in individual cells, and our system allowed the exchange of solutions without exposing the cells to high concentrations of HCO_3_^–^ and KCl, and without changes in the osmolarity of the solutions.

Our results are consistent with others’ observations that the sAC and PKA pathways are important in regulating sperm capacitation in many species and that these pathways act, at least in part, by regulating pH_*i*_ (Puga Molina et al., 2017; Chavez et al., 2019; Matamoros-Volante and Trevino, 2020; Balbach et al., 2021). Carlson et al. showed no changes in pH_*i*_ induced by HCO_3_^–^ in mouse sperm. However, those experiments only tested the effect of HCO_3_^–^ during the first 30–60 s of HCO_3_^–^ application (Carlson et al., 2007). Notably, the changes in pH_*i*_ that we measured after applying HCO_3_^–^ occurred in the same temporal window as the activation of PKA (1–2 min) (Buffone et al., 2014).

Our observation that the HCO_3_^–^-induced change in pH_*i*_ was inhibited by a specific sAC inhibitor (Balbach et al., 2020, 2021) suggests that the main mechanism of pH_*i*_ increase is through the sAC/cAMP/PKA pathway. This increase in pHi might further activate sAC, as Xie et al. showed that sAC activity increases upon intracellular alkalinization (Xie et al., 2006). Further work is needed to determine whether PKA solely regulates CatSper through pH_*i*_ or also through other mechanisms. Adonal work is also needed to identify the molecular mechanism responsible for the increase in pH_*i*_ induced by PKA.

## Data Availability Statement

The raw data supporting the conclusions of this article will be made available by the authors, without undue reservation.

## Ethics Statement

The studies involving human participants were reviewed and approved by University in St. Louis Institutional Review Board (protocol #201706077). The patients/participants provided their written informed consent to participate in this study. The animal study was reviewed and approved by Institutional Animal Care and UseCommittee of Washington University in St. Louis (St. Louis, MO) (protocol # 20-0126).

## Author Contributions

JF designed, performed, analyzed data, and interpreted the results. PL designed and analyzed experiments. LP-M designed, performed, analyzed experiments. CMS analyzed data and interpreted the results. JF and LP-M prepared the figures. JF and CMS drafted the manuscript. JF, PL, LP-M, and CMS edited, revised, and approved the final version of the manuscript. All authors contributed to the article and approved the submitted version.

## Conflict of Interest

The authors declare that the research was conducted in the absence of any commercial or financial relationships that could be construed as a potential conflict of interest.

## Publisher’s Note

All claims expressed in this article are solely those of the authors and do not necessarily represent those of their affiliated organizations, or those of the publisher, the editors and the reviewers. Any product that may be evaluated in this article, or claim that may be made by its manufacturer, is not guaranteed or endorsed by the publisher.
