# Supplementary material for: Conserved Mechanism of Bicarbonate-Induced Sensitization of CatSper Channels in Human and Mouse Sperm
Source: Front Cell Dev Biol. 2021 Sep 28;9:733653. doi: 10.3389/fcell.2021.733653 (PMC8505895; doi:10.3389/fcell.2021.733653)
Supplement: Supplementary file 1 [file Data_Sheet_1.pdf]

Supplementary Figures

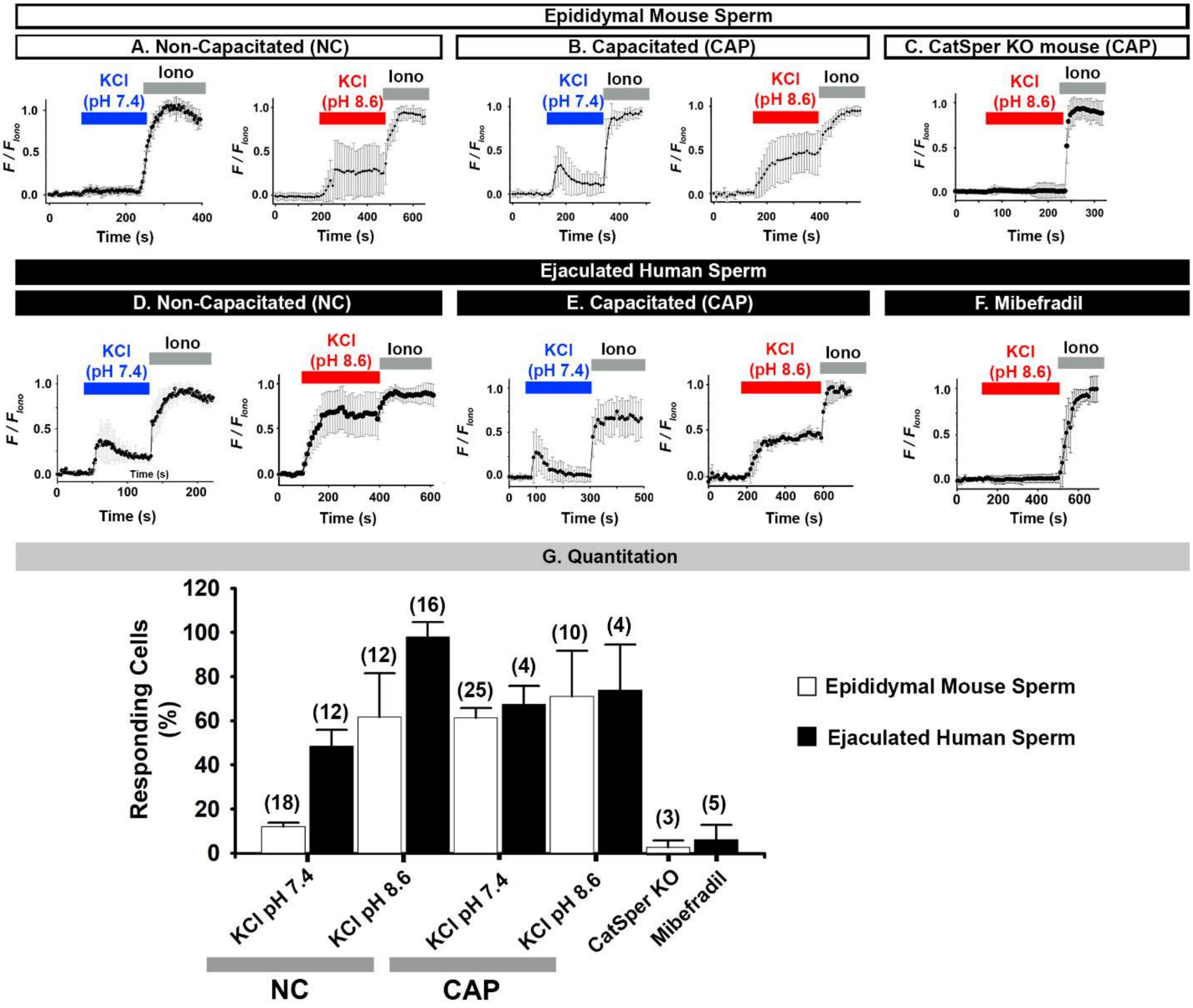

Supplementary Figure 1. Intracellular Ca^{2+} responses to neutral and alkaline depolarization in mouse epididymal and human ejaculated sperm.(A-F) Representative traces of mean Ca^{2+} responses to 50 mM KCl pH 7.4 (Blue) or pH 8.6 (Red) in**(A)** non-capacitated epididymal mouse sperm,**(B)**capacitated epididymal mouse sperm,**(C)**capacitated epididymal sperm from CatSper KO mouse, **(D)** non-capacitated ejaculated human sperm,**(E)**capacitated ejaculated human sperm, and**(F)**ejaculated human sperm after inhibiting CatSper channels with 20 μM Mibefradil. **(G).** Graph of the mean and standard deviation of the percentage of sperm showing increased $[Ca^{2+}]_i$. Iono = 5 mM Ionomycin. Numbers in parentheses are numbers of independent mouse or human samples.

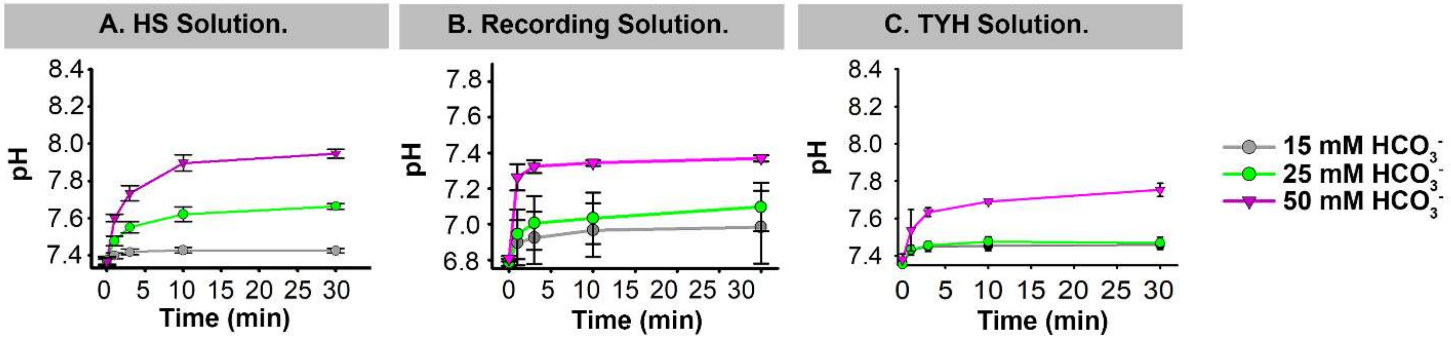

Supplementary Figure 2. Low concentration of HCO_3^- has minimal effect on pH of recording solutions. Mean and standard deviation of pH over time in **(A)** HS solution, in which mouse sperm were recorded, **(B)** Recording solution, in which human sperm were recorded, and **(C) TYH** solution, in which intracellular pH from mouse sperm were recorded.

.
